# Monitoring of Vedolizumab Infusion Therapy (MOVE-IT) Response With Fecal Inflammation Markers, Ultrasound, and Trough Serum Level in Patients With Ulcerative Colitis: Protocol for a Multicentric, Prospective, Noninterventional Study

**DOI:** 10.2196/14335

**Published:** 2019-11-08

**Authors:** Jimmi Cording, Margit Blömacher, Berit Inga Wiebe, Jost Langhorst, Torsten Kucharzik, Andreas Sturm, Stefan Schreiber, Ulf Helwig

**Affiliations:** 1 Kompetenznetz Darmerkrankungen eV Kiel Germany; 2 Klinikum Bamberg Bamberg Germany; 3 Klinikum Lüneburg Lüneburg Germany; 4 DRK Kliniken Berlin | Westend Berlin Germany; 5 University of Kiel Kiel Germany; 6 Medical Practice for Internal Medicine Oldenburg Oldenburg Germany

**Keywords:** ulcerative colitis, vedolizumab, ultrasound

## Abstract

**Background:**

Vedolizumab has been shown to induce clinical remission in patients with active ulcerative colitis. Treatment with anti-integrin vedolizumab leads to clinical remission in 16.9% and clinical response in 47.1% of cases after 6 weeks. However, in clinical practice, no decision to discontinue or continue vedolizumab therapy is made until 14 weeks at the earliest.

**Objective:**

The aim of this study is to develop an algorithm for optimizing vedolizumab administration in patients with moderate-to-severe ulcerative colitis by calculating the probability of clinical response at week 14, on the basis of the data from week 6.

**Methods:**

This is a prospective, single-arm, multicentric, noninterventional, observational study with no interim analyses and a sample size of 35 evaluable patients.

**Results:**

The enrollment started in August 2018 and was still open at the date of submission. The study is expected to complete in September 2020.

**Conclusions:**

The early identification of patients who are responding to an integrin antibody is therapeutically beneficial. At the same time, patients who are not responding can be identified earlier. The development of a therapeutic algorithm for identifying patients as responders or nonresponders can thus help prescribing physicians avoid ineffective treatments and stop these very early.

## Introduction

### Background

Ulcerative colitis (UC) belongs to the group of chronic inflammatory bowel diseases (IBD), with a chronic recurrent course of disease. Vedolizumab has been shown to induce clinical remission in patients with active UC [1]. Treatment with anti-integrin vedolizumab leads to clinical remission in 16.9% and clinical response in 47.1% of cases after 6 weeks [1]. However, in clinical practice no decision to discontinue or continue vedolizumab therapy is made until 14 weeks at the earliest. Sometimes, clinical response could be improved by an additional infusion at week 10. The decision to perform this infusion has not been defined yet.

Early identification of patients responding to an anti-integrin antibody would result in a therapeutic benefit, whereas patients who would not respond could be identified earlier than usual. This approach would lead to a safer anti-integrin antibody application; consequently, this would lead to an increased penetration rate of biological treatment in IBD patients.

### Objective

This study aims to create a decision algorithm for the optimized use of vedolizumab. The algorithm is based on measurements of early changes in noninvasive clinical markers, such as fecal calprotectin, intestinal ultrasound (IUS), and drug levels.

In interventional pivotal studies, the partial Mayo score, as used in the GEMINI study [1,2], is often regarded as the gold standard.

Here, we also seek to use IUS to determine the course of IBD disease. In the last decade, IUS has emerged as an important imaging modality in the diagnosis of Crohn disease (CD), as well as for monitoring disease progression, and in the therapeutic response to CD and UC. The technique is of growing importance in IBD [3].

## Methods

### Trial Design

The study is being carried out in conformity with the German Medicinal Products Act (*Arzneimittelgesetz*, AMG) and is a noninterventional study in accordance with the Medicinal Products Act (§ 4 section 23 p. 3 AMG). The study is designed as a prospective, single-arm, multicentric, noninterventional, observational study, with no interim analyses and a sample size of 35 evaluable patients, for which 50 patients need to be recruited.

### Outcomes

#### Primary

The aim of the study is to show that a change in selected parameters—positive drug levels ≥24 µg/mL, fecal calprotectin ≥50%, and changes in abdominal ultrasound properties (≥25% reduction in wall thickness)—compared with the baseline value at week 6 are reliable predictors of clinical response at week 14.

#### Trough Serum Level 

We assume that early measurable positive trough level might have a predictive value for the clinical response. Data from studies involving vedolizumab showed predictive interpretation on trough serum levels of vedolizumab. It was shown that 87.67% of patients at week 6 responded in a trough-level–dependent manner. In addition, studies on antitumor necrosis factor (TNF) agents have shown that the early drug level is important to predict response to therapy [1,4].

#### Secondary

To predict the probability of a clinical response to a therapy with the integrin antibody vedolizumab at week 22, we monitored the elevation of the fecal calprotectin level in week 6, of the abdominal ultrasound, and the drug level. In addition, it is being investigated whether other stool markers (lactoferrin, S100A12, or Polymorphonuklear-(PMN) elastase) can be used as predictors for a clinical response to a vedolizumab therapy.

In addition to the primary study goal, the following secondary outcomes are being analyzed:

- The different markers (combinations of markers), regarding their correlation with the clinical response (eg, receiver operating characteristic curves or chi-square);
- At which time point a 50% reduction of fecal inflammation markers and ultrasound are reliable predictors of response (other than at 6 weeks);
- Whether antidrug antibodies (ADA) formation at week 6 correlates with clinical response, at least in anti-TNF treatment, and is an important marker [5]; the presence of ADA will be determined using the Vedolizumab free ADA enzyme-linked immunosorbent assay kit (Immundiagnostik AG);
- Whether ADA and vedolizumab levels correlate with any levels of fecal inflammation markers—calprotectin assay (TechLab, Inc);
- Ultrasound (which stool marker has the best sensitivity);
- Whether the following parameters are associated with clinical response—serum C-reactive protein (CRP) level, number of leukocytes, number of thrombocytes, hemoglobin level, and if applicable, ferritin level;
- The rates of adverse events are documented and evaluated; and
- The therapy maintenance is measured by at a follow-up visit at week 52.

### Statistics

#### Statistical Analysis

The hypothesis for the primary endpoint of predicting clinical response at week 14 by at least 2 improved markers (drug level, intestinal ultrasound, and calprotectin level) at week 6 is as follows:

H0: The probability of response P_1_=n_11_/n_1+_ and P_2_=n2_21_/n_2+_ is the same for both groups (improved markers vs nonimproved markers).

H_0_: P_1_=P_2_

vs

H1: The probability of response is not the same.

H_1_: P_1_≠P_2_

The sample size calculation will be done with a chi-square test.

The secondary endpoints will be analyzed with suitable statistical methods, such as receiver operating characteristic curves or rank correlation for correlations. To analyze parameters associated with clinical response, a regression analysis will be performed. The analysis of adverse events will be done with appropriate descriptive methods.

There will be no interims analysis; there will be only 1 analysis at the end of the study (Table 1).

**Table 1:** Calculation of response rate to vedolizumab infusion therapy.

	Clinical response (week 14)		
At least 2 improved markers (week 6)	Yes	No	Total
Yes	n_11_	n_12_	n_1+_
No	n_21_	n_22_	n_2+_
Total	n_+1_	n_+2_	N/A^a^

^a^Not applicable.

#### Sample Size Calculation

The following assumptions were used to calculate the sample size (Table 2):

- The rate of clinical response at week 14 is 0.57 [6].
- The assumptions for clinical response rates at week 14 for the changed markers at week 6 are that 80% of patients with at least two improved markers at week 6 will have a clinical response at week 14, and 25% of patients who have less than 2 improved markers at week 6 will have a clinical response at week 14.

The clinical response in week 14 is measured with the partial Mayo score:

- Reduction of <2 points (clinical response)
- Partial Mayo score of ≤2 points (remission)

These assumptions result in a sample size of 36 patients (calculated with the R function Basic Functions for Power Analysis [pwr] chi-square test, significance level=0.05, and power=0.9). With a dropout rate of 35%, the sample size is 50 patients.

**Table 2:** Sample size calculation based on a clinical response rate of 0.57.

	Clinical response (week 14)	
At least 2 improved markers (week 6)	Yes	No
Yes	p_11_=0.8	p_12_=0.2
No	p_21_=0.25	p_22_=0.75
Total	p_2+1_=0.57	p_+2_=0.43

### Definition of Study Population

The primary and secondary evaluation criteria are assessed according to the intention-to-treat principle (ITT). The corresponding collective includes all patients included in the study, regardless of possible protocol violations (eg, study terminations). In addition to the ITT analyses, sensitivity analyses are being carried out according to the per-protocol principle. Relevant protocol violations that lead to exclusion from the per-protocol collective are defined in the statistical analysis plan.

### Selection of Study Centers

All study centers are part of the German IBD Study Group, and they are chosen according to their main area of focus and their experience in the treatment of UC. Regarding the results of the IUS examinations, no differences in the diagnostic quality of IUS measurements were found among gastroenterologists [7,8]. By signing the investigator agreement, each study center selected confirms its fulfilment of all formal requirements for inclusion in the study and guarantees its compliance with data privacy laws and any other regulations pertaining to the execution of this observational study.

### Participant Criteria

The inclusion criteria for the study include (1) clinically and endoscopically confirmed diagnosis of UC (3 months before participation in the study); (2) secured disease by increased fecal calprotectin ≥100 μg/g and/or endoscopic score : Ulcerative Colitis Endoscopic Index of Severity ≥3, 3 weeks before the baseline visit; (3) ultrasound detectable disease; sonographic sign of active inflammation, determined by the bowel wall thickness >4 mm (sigmoideum), >3 mm (colon); (4) if an independent treatment with vedolizumab according to the routine medical practice is done, there should a break of at least 12 weeks between the end of the treatment and the beginning of the participation in the study; (5) age ≥18 years and <80 years; (6) signed consent form; (7) start of a study-independent vedolizumab therapy according to medical practice; (8) sufficient German language communication skills; and (9) ability of the patient to understand the nature, significance, and scope of the clinical trial and make an independent decision on the basis of this knowledge.

The exclusion criteria include (1) pregnancy and lactation; (2) off-label treatment with vedolizumab; (3) contraindications for treatment with vedolizumab (according to product information); (4) ileostoma or ileoanal pouch; (5) infectious colitis (eg, *Clostridium difficile* colitis and *Cytomegalovirus* colitis); (6) obesity ≥grade I (body mass index >30): insufficient, sonographic intestinal wall imaging; (7) proctitis; (8) participation in an intervention study within the last 30 days before the start of the vedolizumab therapy; and (9) other medical reasons.

### Study-Specific Interventions

No medical interventions are performed in the course of the study other than those required by the standard medical procedure. When taking routine blood samples, vedolizumab serum levels and anti-vedolizumab antibody levels should also be monitored, if possible. Only the natural progress of the disease in UC patients is monitored and evaluated.

### Schedule of Visits

There are no defined study visits. In the course of the study, the only clinical and laboratory data recorded are those corresponding to the standard medical procedure. Data are recorded in the following observational weeks: baseline/screening, 6, (10, optional), 14, 22, and 52. Deviations of ±5 days from this documentation schedule fall within the scope of the study protocol. The period until the next examination is subsequently shortened or lengthened accordingly to compensate for deviations and maintain the examination rhythm.

The following data are recorded at the initial screening examination: date of consent, screening date, inclusion and exclusion criteria, personal information (date of birth, sex, height, weight, and smoker status), date of initial UC diagnosis, first symptoms, duration of acute symptoms (in days), Montreal classification, and information regarding previous medication (anti-TNF, aminosalicylates, budesonide, systemic corticosteroids, and azathioprine).

During the follow-up visits (baseline, weeks 6, 14, and 22), data on the following parameters are collected: current medication (vedolizumab [time and dose], aminosalicylate, budesnoide, systemic corticosteroids, and azathioprine); partial Mayo score; laboratory tests (hemoglobin, CRP, leukocytes, calprotectin, lactoferrin, PMN elastase, S100A12, vedolizumab trough serum levels, anti-vedolizumab antibodies); and IUS parameters.

At week 10 (optional visit), current medication, partial Mayo score, laboratory tests (hemoglobin, CRP, and leukocytes), current disease activity, notification of serious adverse event/adverse drug reactions events, and special situations are reported. A week 10 infusion is approved in Germany, and it cannot be prevented in an observational study. We assume that this infusion at week 10 will have no influence on the overall result (nor on our predictability). In addition, the intestinal ultrasound is measured. In the follow-up visit (week 52), the maintenance of the therapy is assessed by determining the partial Mayo score.

### Documentation

Data are recorded using case report forms (CRFs). The investigator is responsible for the timely, correct, complete, and legible recording of study data in the CRF and confirms recording by signature. CRFs are completed with a black ballpoint pen. Corrections are documented as follows: The wrong entry is crossed out with a single line, and corrections are entered next to the crossed-out text and verified by date and initials, stating the reason for the change, if necessary. Instructions for use (entry and corrections) are included in each CRF. Source data, according to the International Conference on Harmonization of Technical Requirements for Registration of Pharmaceuticals for Human Use E6 guideline on good clinical practice (GCP), are original documents in patient files, as well as doctors’ letters, certified copies of original records, and laboratory printouts. Study data are to be recorded from patient files.

### Patient Identification

All patient data are pseudonymized. Each patient will be clearly identified by a patient identification number assigned at each study center. The investigator will keep a patient identification list, documenting the patient identification number with the patient’s full name, date of birth, sex, and date of informed consent.

The patient identification list is part of the investigator file, and it will remain at the site. The patient identification number comprises a 2-digit clinic number, as well as a running 2-digit number of recruited patients per study site.

### Trial

#### Start of Patient Participation

Any patient with a clinically and endoscopically confirmed diagnosis of UC and qualified for vedolizumab treatment according to routine medical practice is a potential study candidate. All potential candidates who come to the attention of the investigator will be informed regarding the possibility of participating in the study.

#### End of Patient Participation

The observation of each patient ends according to the schedule with the last study visit. A patient’s participation in the study will be terminated prematurely if at least one of the following criteria is met: (1) withdrawal of informed consent, (2) termination of vedolizumab treatment, (3) lack of medical justification for further participation in the study, (4) premature termination of the complete trial, or (5) subsequent discovery that not all inclusion criteria are met and/or that any exclusion criteria are met.

#### Trial Duration/End of Trial

The recruiting phase has a planned duration of 24 months. The observational phase has a planned duration of 52 weeks. The complete trial is considered to have ended after all queries from the study coordination center have been answered by each individual study center, at the latest, 4 months after the last visit of the last patient.

Study centers that grossly violate the AMG, data protection regulations, or the GCP guidelines can be excluded from the further recruitment and observation of study patients. Premature termination of the study as a whole will be taken into consideration if ethical or scientific justification for the trial is compromised or no longer valid, errors or violations significantly compromise the scientific integrity of the data collected for the study with regard to the study aims, or the requirements for a successful execution of the study are no longer fulfilled for other reasons. The principal investigator will consult the corresponding biometrician regarding any possible premature termination of the trial. The minutes of the aforementioned consultation meeting will be recorded and subjected to the approval of both parties. Any decision regarding the premature termination of the trial will be taken jointly by the principal investigator and the corresponding biometrician.

#### Data Quality Assurance

Upon receipt at the study coordination center, CRF will be checked for completeness and consistency (in-house review). Queries will be generated for missing or implausible entries and sent to the corresponding study centers. After the clarification of implausible entries and completion of missing data, CRF will be handed over to the corresponding data management department for data entry.

#### Quality Control and Assurance

The principal investigator and/or auditors designated by the principal investigator are entitled to conduct audits at the study centers and any other facilities participating in the study. They are entitled to inspect and review all study-relevant documents. This right also applies to regulatory inspectors.

#### Ethical and Regulatory Aspects

The study is conducted in compliance with the current version of the Declaration of Helsinki (October 2013, Fortaleza, Brazil). This study cannot begin before approval has been obtained from the corresponding ethics committee. Before inclusion in the study, the investigator will inform each patient about the nature, significance, risks, and scope of the study, as well as the patient’s right to withdraw from the study at any time without prejudice. An informed consent form is handed to the patient, describing the study in nonscientific and generally understandable language. Each patient must consent to study participation in writing. The patient must be provided with adequate time to decide with the opportunity to ask any questions before the consent form is signed.

In accordance with AMG, § 40 Abs. 2a, patients are informed that the data related to their disease will be stored with a pseudonym and analyzed for scientific purposes. Patients must consent to the use of their pseudonymized data in writing. Informed consent forms are to be signed and dated by the patient and the treating physician.

This clinical study is carried out in conformity with the requirements of the current German Medicinal Products Act, as well as all applicable legal provisions regarding data protection and the GCP guidelines. The general notification requirement as per § 67 AMG will be complied with.

## Results

The enrollment started in August 2018 and was still open at the date of submission. The study is expected to complete in August 2020.

## Discussion

### Rationale for the Trial

In this study, a prospective study approach was chosen, as the probability of a clinical response at week 14 is to be calculated on the basis of the data from week 6. A single cohort is needed to answer this question. It is not necessary to compare groups with the same structure.

### Justifications for Trial Design

To achieve a higher representativeness of the study statement for the population as a whole, the study will be conducted nationwide and multicentrally, with specially selected study sites. The treatment and diagnosis do not follow a predefined test plan, but they exclusively follow the medical practice. A vedolizumab infusion at week 10 is approved in Germany, and this cannot be prevented in an observational study. We assume that this infusion at week 10 will have no influence on the overall result (nor on our predictability). This noninterventional approach is intended to strengthen the representativeness of the study statement for everyday medical practice, as no reduction in the dispersion of the target parameters is achieved through different experimental approaches.

### Trough Serum Level

Trough serum levels above 24 µg/mL of vedolizumab at week 6 are associated with clinical remission and clinical response [1]. Early low trough serum level and antibody detection toward the therapeutic drug antibody are at least documented for TNF-alpha antibodies, and they are associated with a poor response [5]. Therefore, an early measurable high trough serum level and the absence of antibodies might have a predictive value on the clinical response [1].

### Stool Marker

Fecal calprotectin reflects the mucosal inflammation status. The good correlation between high fecal calprotectin and mucosal inflammation is described previously [9,10]. We assume a clear activity, measured by fecal calprotectin and/or endoscopic score. Therefore, the Mayo score was chosen as the inclusion criterion. Furthermore, there is no doubt, that fecal calprotectin is diminished during a valuable therapeutic response. At least in the UC trial, calprotectin was significantly diminished in the verum arm versus placebo arm [1]. The median calprotectin level showed a reduction of 50% (from 1000 µg/g to 500 µg/g) [1]. Different stool markers have diverse sensitivity [11]. Therefore, a different determination of stool markers might be useful to distinguish between responders and nonresponders. The prediction of response is shown for fecal calprotectin [12], lactoferrin [13], and S100A12 [11,14].

### Intestinal Ultrasound

The detection of intestinal inflammation by ultrasound is a well-established but underused method [3]. Recently, we have shown that the reduction of ultrasound features correlates with clinical response in CD [7,8]. Similar data were presented at the United European Gastroenterology week 2017 in Barcelona for UC [15]. Furthermore, early response was seen in UC by rectal ultrasound [16].

This early monitoring of response study aims to achieve a more thorough understanding of therapeutic development in patients with moderate-to-severe UC, receiving regular doses of vedolizumab, by developing an algorithm for optimizing vedolizumab administration.

## Abbreviations

ADA: antidrug antibodies
AMG: Arzneimittelgesetz, German Medicinal Products Act
CD: Crohn disease
CRF: case report form
CRP: C-reactive protein
GCP: good clinical practice
IBD: inflammatory bowel disease
ITT: intention-to-treat
IUS: intestinal ultrasound
PMN: Polymorphonuklear
TNF: tumor necrosis factor
UC: ulcerative colitis

## References

[1] Feagan BG, Rutgeerts P, Sands BE, Hanauer S, Colombel JF, Sandborn WJ, van Assche G, Axler J, Kim HJ, Danese S, Fox I, Milch C, Sankoh S, Wyant T, Xu J, Parikh A, GEMINI 1 Study Group (2013). Vedolizumab as induction and maintenance therapy for ulcerative colitis. N Engl J Med.

[2] de Vos M, Dewit O, D'Haens G, Baert F, Fontaine F, Vermeire S, Franchimont D, Moreels T, Staessen D, Terriere L, Cruyssen BV, Louis E, Behalf of BIRD (2012). Fast and sharp decrease in calprotectin predicts remission by infliximab in anti-TNF naïve patients with ulcerative colitis. J Crohns Colitis.

[3] Bryant RV, Friedman AB, Wright EK, Taylor KM, Begun J, Maconi G, Maaser C, Novak KL, Kucharzik T, Atkinson NS, Asthana A, Gibson PR (2018). Gastrointestinal ultrasound in inflammatory bowel disease: an underused resource with potential paradigm-changing application. Gut.

[4] Schulze H, Esters P, Hartmann F, Stein J, Christ C, Zorn M, Dignass A (2018). A prospective cohort study to assess the relevance of vedolizumab drug level monitoring in IBD patients. Scand J Gastroenterol.

[5] Cornillie F, Hanauer SB, Diamond RH, Wang J, Tang KL, Xu Z, Rutgeerts P, Vermeire S (2014). Postinduction serum infliximab trough level and decrease of C-reactive protein level are associated with durable sustained response to infliximab: a retrospective analysis of the ACCENT I trial. Gut.

[6] Amiot A, Grimaud J, Peyrin-Biroulet L, Filippi J, Pariente B, Roblin X, Buisson A, Stefanescu C, Trang-Poisson C, Altwegg R, Marteau P, Vaysse T, Bourrier A, Nancey S, Laharie D, Allez M, Savoye G, Moreau J, Gagniere C, Vuitton L, Viennot S, Aubourg A, Pelletier A, Bouguen G, Abitbol V, Bouhnik Y, Observatory on Efficacyof Vedolizumab in Patients With Inflammatory Bowel Disease Study Group, Groupe d'Etude Therapeutique des Affections Inflammatoires du tube Digestif (2016). Effectiveness and safety of vedolizumab induction therapy for patients with inflammatory bowel disease. Clin Gastroenterol Hepatol.

[7] Kucharzik T, Wittig BM, Helwig U, Börner N, Rössler A, Rath S, Maaser C, TRUST Study Group (2017). Use of intestinal ultrasound to monitor Crohn's disease activity. Clin Gastroenterol Hepatol.

[8] Kucharzik T, Wittig BM, Helwig U, Roessler A, Rath S, Maaser C (2015). Bowel ultrasound is useful in Crohn's disease monitoring: analysis from the TRUST study in Germany. J Crohns Colitis.

[9] Reinisch W, Bressler B, Curtis R, Parikh A, Yang H, Rosario M, Røseth A, Danese S, Feagan B, Sands BE, Ginsburg P, Dassopoulos T, Lewis J, Xu J, Wyant T (2019). Fecal calprotectin responses following induction therapy with vedolizumab in moderate to severe ulcerative colitis: a post Hoc analysis of GEMINI 1. Inflamm Bowel Dis.

[10] Walsham NE, Sherwood RA (2016). Fecal calprotectin in inflammatory bowel disease. Clin Exp Gastroenterol.

[11] Barnes EL, Burakoff R (2016). New biomarkers for diagnosing inflammatory bowel disease and assessing treatment outcomes. Inflamm Bowel Dis.

[12] Langhorst J, Boone J, Lauche R, Rueffer A, Dobos G (2016). Faecal Lactoferrin, Calprotectin, PMN-elastase, CRP, and white blood cell count as indicators for mucosal healing and clinical course of disease in patients with mild to moderate ulcerative colitis: post Hoc analysis of a prospective clinical trial. J Crohns Colitis.

[13] Langhorst J, Elsenbruch S, Koelzer J, Rueffer A, Michalsen A, Dobos GJ (2008). Noninvasive markers in the assessment of intestinal inflammation in inflammatory bowel diseases: performance of fecal lactoferrin, calprotectin, and PMN-elastase, CRP, and clinical indices. Am J Gastroenterol.

[14] Däbritz J, Langhorst J, Lügering A, Heidemann J, Mohr M, Wittkowski H, Krummenerl T, Foell D (2013). Improving relapse prediction in inflammatory bowel disease by neutrophil-derived S100A12. Inflamm Bowel Dis.

[15] Maaser C, Petersen F, Helwig U, Rössler A, Lang D, Rath S, Kucharzik T (2017). Bowel ultrasound is useful in disease monitoring of ulcerative colitis patients: first analysis from the TRUST&UC study in Germany. https://tinyurl.com/yxon5rlh.

[16] Ellrichmann M, Bethge J, Zeissig S, Brandt B, Arlt A, Nikolaus S, Schreiber S, Lankau J (2015). Changes of the colonic wall structure precede mucosal healing in patients with acute inflammatory bowel disease undergoing anti-integrin therapy - preliminary results of a prospective study. United Eur Gastroenterol J.

